# Association between 25(OH) vitamin D and schizophrenia: shared genetic correlation, pleiotropy, and causality

**DOI:** 10.3389/fnut.2024.1415132

**Published:** 2024-12-13

**Authors:** Guo-Wei Rong, Xiao-Min Li, Hui-Min Lu, Ming-Zhu Su, Yi Jin

**Affiliations:** ^1^The Wujin Clinical College of Xuzhou Medical University, Changzhou, Jiangsu, China; ^2^Department of Orthopedics, Wujin Hospital Affiliated with Jiangsu University, Changzhou, Jiangsu, China; ^3^Jiangsu Province Key Laboratory of Anesthesiology, Xuzhou Medical University, Xuzhou, Jiangsu, China; ^4^Department of Pharmacy, Wujin Hospital Affiliated with Jiangsu University, Changzhou, Jiangsu, China; ^5^Department of Outpatient and Emergency, First Affiliated Hospital of Soochow University, Suzhou, Jiangsu, China; ^6^Department of Good Clinical Practice, Wujin Hospital Affiliated with Jiangsu University, Changzhou, Jiangsu, China

**Keywords:** vitamin D, schizophrenia, genetic overlap, Mendelian randomization, genome-wide association study

## Abstract

**Background:**

This study delves into the complex interplay between genetics, 25-hydroxyvitamin D (25OHD), and schizophrenia (SCZ). It leverages extensive sample data derived from Genome-Wide Association Studies (GWAS) to uncover genetic correlations.

**Methods:**

Employing Linkage Disequilibrium Score Regression (LDSC) and S-LDSC, this study investigates genetic connections between 25OHD and SCZ. It examines Single Nucleotide Polymorphism (SNP) heritability in specific tissues and incorporates diverse immune cell datasets for genetic enrichment analysis. Local genetic correlations were analyzed using HESS software, and pleiotropy analysis identified shared genetic loci in brain tissues. Hyprcoloc analysis was used to explore shared genetic factors between 25OHD, immune cells, and SCZ, complemented by a bidirectional Mendelian Randomization (MR) to probe potential causal links.

**Results:**

We identified a significant negative genetic correlation between 25OHD levels and SCZ. PLACO analysis revealed 35 pleiotropic loci with strong enrichment in brain regions, particularly the cerebellum, frontal cortex, and hippocampus. Eight loci (1p34.2, 2p23.3, 3p21.1, 5q31.2, 12q23.2, 14q32.33, 16p13.3, and 16q24.3) exhibited strong colocalization, highlighting potential drug targets. Gene and tissue enrichment analyses emphasized neurological and immune-related mechanisms, including hyaluronan metabolism. Bidirectional MR analysis supported a causal effect of SCZ on 25OHD levels.

**Conclusion:**

Our study identifies NEK4 as a potential therapeutic target and highlights the involvement of hyaluronan metabolism in the genetic association between 25OHD and SCZ. These findings provide valuable insights into shared genetic pathways, immune-related connections, and causal interactions in the context of SCZ.

## 1 Introduction

Schizophrenia (SCZ) is a complex mental disorder that affects about 21 million people worldwide (1). Although the pathogenesis of SCZ is unclear, SCZ has been shown to have etiological links to events early in life, at birth and even *in utero* (2, 3). Epidemiological evidence has demonstrated that a person's risk of developing SCZ is influenced by factors such as childhood trauma, environmental displacement, social isolation, advancing urbanization, and substance abuse (4). In more detail, the prevalence is higher in children born in winter, living at high latitudes and growing up in cities (5–7). All of these factors are associated with reduced sunlight exposure, so low 25OHD levels are considered a risk factor for SCZ (8).

Most vitamin D is synthesized in the skin with the help of sunlight, and only a small portion is taken in through the diet (9). The amount of vitamin D synthesized depends on age, skin color, season, duration of sun exposure, and latitude of residence (10). Vitamin D3 is first hydroxylated in the liver to produce 25OHD, and then the physiologically active 1, 25 dihydroxyvitamin 2D (1, 25OH2D) is produced in the kidneys, so serum 25OHD levels are the best parameter to represent vitamin D levels (11). Recent studies have found that 25OHD, in addition to its involvement in the regulation of calcium and phosphorus metabolism in the body and the maintenance of bone health, is potentially linked to psychiatric disorders such as SCZ, depression and anxiety (12, 13). The association may be predicated on the fact that both 25OHD and 1, 25OH2D can cross the blood-brain barrier, and that vitamin D receptors and vitamin D metabolizing enzymes are present in human and rodent brain tissue (14–17). 25OHD increases the number of mature macrophages, raises the expression levels of CD36 and PPAR-γ in the brain, benefits neurological recovery and promotes the clearance of blood masses in mice after cerebral hemorrhage (18). Imaging studies have found a significant positive correlation between 25OHD levels and hippocampal volume in patients with SCZ (19). 25OHD has an important protective effect on the hippocampus.

Low levels of 25OHD in fetuses or newborns increase risk of SCZ (20). A Danish case-control study (*N* = 868) found a significant association between low 25OHD levels in newborns and the risk of SCZ later in life (21). Another case-control study (*N* = 2,602) with a larger sample size replicated the association of neonatal 25OHD deficiency with a significantly increased risk of SCZ (22). Such evidence points to the hypothesis that maternal 25OHD deficiency is a risk factor for SCZ (8). The meta-analysis found that people with SCZ were more likely to be 25OHD deficient (23, 24). However, this does not confirm an independent association: people with SCZ generally have reduced exercise and diet and do not spend enough time in the sun, which are known to contribute to lower 25OHD levels. In conclusion, controversy with regard to the true link between 25OHD and SCZ has been unsettled.

**Figure 1:**
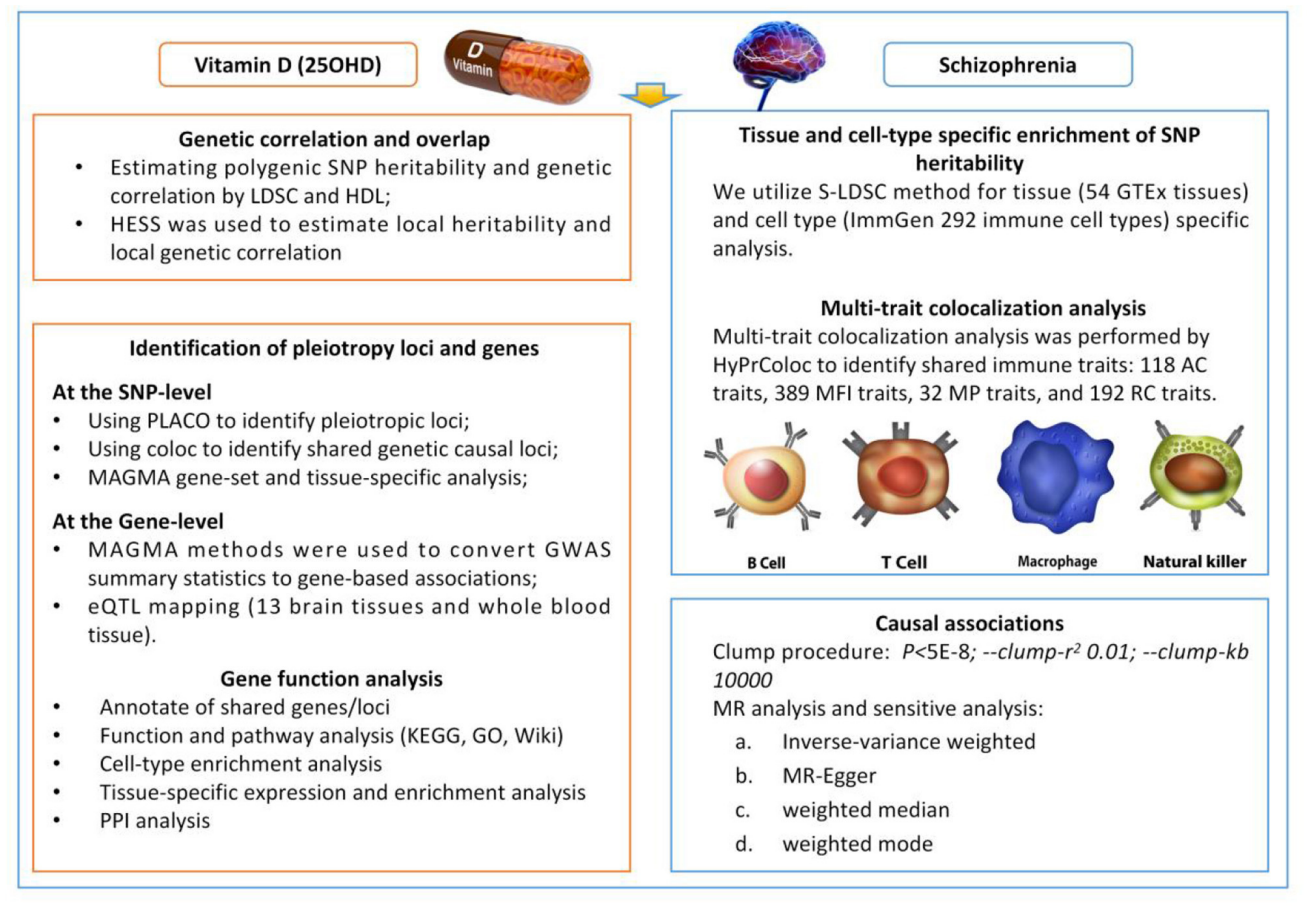
Overview of statistical analyses performed in the study. SNP, Single Nucleotide Polymorphism; LDSC, Linkage Disequilibrium Score Regression; HDL, high-definition likelihood; HESS, Heritability Estimation from Summary Statistics; PLACO, Protein–Ligand Affinity by Comparable Co-Evolution; MAGMA, Multi-marker Analysis of GenoMic Annotation; GWAS, Genome-Wide Association Study; eQTL, Expression Quantitative Trait Locus; S-LDS, Stratified Linkage Disequilibrium Score.

Traditional observational epidemiologic studies have many limitations in establishing causal relationships between disease exposures and outcomes, including reverse causal associations with potential confounders that bias results and render them unreliable (25). Well-designed randomized controlled trials are the gold standard for determining the association between 25OHD levels and the risk of SCZ, but are difficult to conduct for large samples followed over decades. By integrating genetic epidemiological methods such as Mendelian randomization (MR), pleiotropy, and genetic correlation analyses, potential associations in complex phenotypes can be explored more reliably. This approach has demonstrated its advantages in research across multiple fields (26–28). Numerous studies have shown that 25OHD is involved in evolutionary processes associated with SCZ pathogenesis, and genetic variation may influence such neurobiological processes directly or indirectly through 25OHD levels. Therefore, it is important to further investigate potential overlapping genetic structures. Based on these factors, we systematically evaluated the complex relationship between 25OHD and SCZ in terms of genetic correlation, pleiotropy, and causality (Figure 1).

## 2 Materials and methods

### 2.1 Study design

In order to assess the complex genetic relationship between 25OHD and SCZ, this present study evaluated the genetic correlation between them using the linkage disequilibrium score regression (LDSC) method, and identified the corresponding pleiotropic loci and genes between the two phenotypes using the composite null hypothesis (PLACO). Finally we assessed the causal associations between 25OHD and SCZ using two-sample bidirectional MR method.

### 2.2 25OHD data

25OHD data were obtained from a genome-wide association study (GWAS) of 25OHD levels in 401,460 white British samples (29). The study measured 25OHD levels in twice-collected blood samples in a linear mixed model GWAS of standardized log-transformed 25OHD levels with covariates including age, sex, season, and vitamin D supplementation. A meta-analysis of GWAS results from 42,274 European samples was also performed, resulting in the detection of 138 conditionally independent Single Nucleotide Polymorphisms (SNPs, 63 novel) and an estimated SNP heritability of 16.1% for the 25OHD.

### 2.3 Schizophrenia data

The GWAS summary data for SCZ were derived from a two-stage genome-wide association study involving 55,365 European SCZ patients and 219,884 control individuals (30). This study reported 287 common variants associated with SCZ. In our study, we selected only European ancestry samples to align with the 25OHD dataset. In each individual cohort, association tests were performed using an additive logistic regression model with PLINK, and the results were finally meta-analyzed using an inverse variance-weighted fixed-effects model.

### 2.4 Linkage disequilibrium score regression

The Linkage Disequilibrium Score Regression (LDSC) method was used to analyze genetic correlations, showing how different traits might share common genetic factors (31). This method calculates the extent of shared genetic structures between traits, which helps identify their potential genetic links. LD scores in LDSC were calculated using European ancestry samples from the 1,000 Genomes Project and the HapMap3 project as reference panels (32). Additionally, high-definition likelihood (HDL) analysis was performed to increase the precision of these estimates and enhance statistical power (33), using a reference panel from approximately 300,000 individuals with European ancestry in the UK Biobank. For SNPs, stringent quality control measures were implemented: (1) Exclusion of non-biallelic SNPs and SNPs with ambiguous strand information; (2) Exclusion of SNPs without rs tags; (3) Removal of duplicate SNPs or those not present in the 1,000 Genomes Project or with mismatched alleles; (4) SNPs located within the major histocompatibility complex (chr6: 28.5–33.5 Mb) region were excluded from LDSC analysis due to their complex LD structure. (5) SNPs with a minor allele frequency (MAF) >0.01 were retained. Missing data were handled by excluding SNPs with incomplete information to prevent potential biases in the analysis.

### 2.5 Stratified linkage disequilibrium score regression

We used Stratified Linkage Disequilibrium Score Regression (S-LDSC) to explore whether genetic heritability for 25OHD and SCZ is enriched in specific cell types and tissues, such as the brain or immune cells. This method helps determine if certain tissues or cell types show stronger genetic influences related to these traits, providing insights into where these genetic effects are most active. We obtained human tissue data from GTEx consortium, including 54 different tissues (comprising 13 distinct brain tissues) (34), and obtained data on 292 immune cell types from the ImmGen consortium (35). After adjusting for baseline models and all gene sets, we used the *p*-values of the regression coefficient z-scores to evaluate the significance of SNP heritability enrichment estimates in each tissue and immune cell type.

### 2.6 Local genetic correlation analysis

We applied the Heritability Estimation from Summary Statistics (HESS) to estimate local genetic correlation and assessed the genetic overlap in distinct phenotypes within locally independent genomic regions (36). This approach helps identify areas in the genome where genetic overlap between 25OHD and SCZ may be concentrated, providing more detailed insights into specific genetic regions. A total of 1,702 independent LD regions were included in the analysis. In the first step, HESS computed the eigenvalues of the LD matrix, as well as the squared projection of the GWAS effect size vectors onto the eigenvectors of the LD matrix. In the second step, HESS utilized the output from step 1 to estimate local SNP heritability and its standard error. In the third step, HESS used the output from step 2 to obtain estimates of local genetic covariance and their standard errors. The reference dataset was constructed based on the hg19 genome using samples from the 1,000 Genomes European population.

### 2.7 Pleiotropic analysis under composite null hypothesis

SNP-Level PLACO is a novel method used to study the polygenic loci underlying complex traits using only summary-level genotype-phenotype association statistics (28, 37, 38). The purpose of PLACO analysis is to identify genetic regions that may be shared between 25OHD and SCZ. This type of pleiotropy analysis helps us pinpoint genetic loci that are linked to both traits, revealing potential shared genetic foundations and biological mechanisms. Specifically, we computed the square of the Z-scores for each variant and removed SNPs with extremely high Z^2^ (>80). Considering the potential correlation between 25OHD and SCZ, we estimated the correlation matrix of Z-scores. Subsequently, we used a level-α intersection-union test (IUT) method to assess the hypothesis of no pleiotropy. The final *p*-value for the IUT test was determined by the maximum *p*-value between H_0_ and H_1_.

Based on the PLACO results, we further mapped the identified loci to nearby genes to explore the shared biological mechanisms of these polygenic sites. We conducted a Generalized Gene-Set Analysis of GWAS Data (MAGMA) analysis on genes located at or overlapping with the polygenic loci identified based on PLACO output and single-trait GWAS to identify candidate pathways associated with polygenicity and tissue enrichment of polygenic genes (39). Functional Mapping and Annotation (FUMA) using genome-wide association study data were utilized to determine the biological functions of the polygenic loci (40). Pathway enrichment analysis was performed based on a range of pathways using the Molecular Signatures Database (MSigDB) to determine the functions of the mapped genes (41). eQTL analysis incorporated SNP-gene association data from whole blood tissues.

### 2.8 Colocalization analysis

To identify whether shared genetic regions between 25OHD and SCZ are likely due to common causal variants, we conducted Bayesian colocalization analysis using the R package “coloc.” This method helps determine if two traits have a true shared genetic cause at a given locus or if they simply overlap by chance. Colocalization analysis relies on the assumption of a single causal variant, and for each multi-trait locus, it yields posterior probabilities (PP) for five hypotheses: H_0_: Neither of the two traits has a genetic association in this region; H_1_: Only trait 1 has a genetic association in this region; H_2_: Only trait 2 has a genetic association in this region; H_3_: Both traits are associated but have different causal variants; H_4_: Both traits are associated and share a common causal variant. The “coloc.abf” function was employed for colocalization analysis (P_1_ = P_2_ = 1 × 10^−4^, P_12_ = 1 × 10^−5^).

### 2.9 Hypothesis prioritization for multi-trait colocalization analysis

We utilized the Hypothesis Prioritization for Multi-Trait Colocalization (HyPrColoc) method (42) to conduct multi-trait colocalization analysis by integrating GWAS summary statistics for 731 immune cells from the GWAS catalog (ranging from GCST0001391 to GCST0002121) (43). This analysis aimed to underscore the pivotal role of immune cells in the progression of 25OHD and SCZ. The immune cell traits encompassed various aspects, including 118 absolute cell counts (AC) traits, 389 median fluorescence intensity (MFI) reflecting surface antigen levels traits, 32 morphological parameters (MP) traits, and 192 relative cell counts (RC) traits. Notably, MFI, AC, and RC features covered a range of immune cell subsets, such as B cells, CDC, Ts cell maturation stages, mononuclear cells, bone marrow cells, TBNK, and Treg panels. The MP features were specific to CDC and TBNK panels. Primary GWAS analyses for 731 Immune traits were conducted on a cohort of 3,757 individuals of European descent, with covariates including sex, age, and age^2^. Furthermore, a reference panel based on European sequences was leveraged to estimate approximately 22 million SNPs genotyped using high-density arrays.

### 2.10 Mendelian randomization analysis

MR analysis in this study aims to investigate whether there is a potential causal relationship between 25OHD and SCZ. By using genetic variants as instrumental variables (IVs), MR helps estimate causal effects in a way that minimizes bias from other confounding factors. In this study, significant genetic loci are carefully selected, and sensitivity analyses ensure the reliability of the results, even when different assumptions are tested. This approach allows us to infer causality rather than just correlation, making it a powerful tool for understanding complex relationships in genetic data. In our analysis, we employed the clumping procedure in the PLINK software to select independently significant genetic loci as instrumental variables for two traits (*P* < 5 × 10^−8^) (41). The r^2^ threshold for instrumental variables was set at 0.001, with a physical distance of 10,000 kb window. We also calculated the r^2^ and F-statistic for each of them to ensure the strength of the instrumental variables (44) and the formula for calculating the F-statistic is as follows:


$$\begin{array}{l} F=\left(\frac{n-1-k}{k}\right)\left(\frac{r^{2}}{1-r^{2}}\right) \end{array}$$

Here, r^2^ represents the proportion of variance explained by the instrumental variable, n is the sample size, and k is the number of SNPs.

The primary method used for Mendelian randomization (MR) was the Inverse Variance Weighted (IVW) method, which requires IVs to satisfy three assumptions: (1) IVs should be associated with the exposure; (2) IVs should not be associated with confounding factors related to both the exposure and the outcome; (3) The effect of IVs on the outcome is entirely mediated through the exposure. Several sensitivity analyses were conducted. First, the heterogeneity Q test of IVW and MR-Egger was used to detect potential violations of the assumptions through heterogeneity among the individual IVs (45). Second, intercept of MR-Egger was applied to estimate the horizontal pleiotropy, ensuring that genetic variation is independently associated with exposure and outcome (46). Additional analyses using different modeling assumptions and robust MR methods (weighted median and weighted mode) were employed to enhance the stability and robustness of the results.

All statistical analyses were performed using R version 3.5.3 software, and Mendelian randomization analyses utilized the Mendelian Randomization package (47).

## 3 Results

### 3.1 Genetic correlation between 25OHD and SCZ

**Figure 2:**
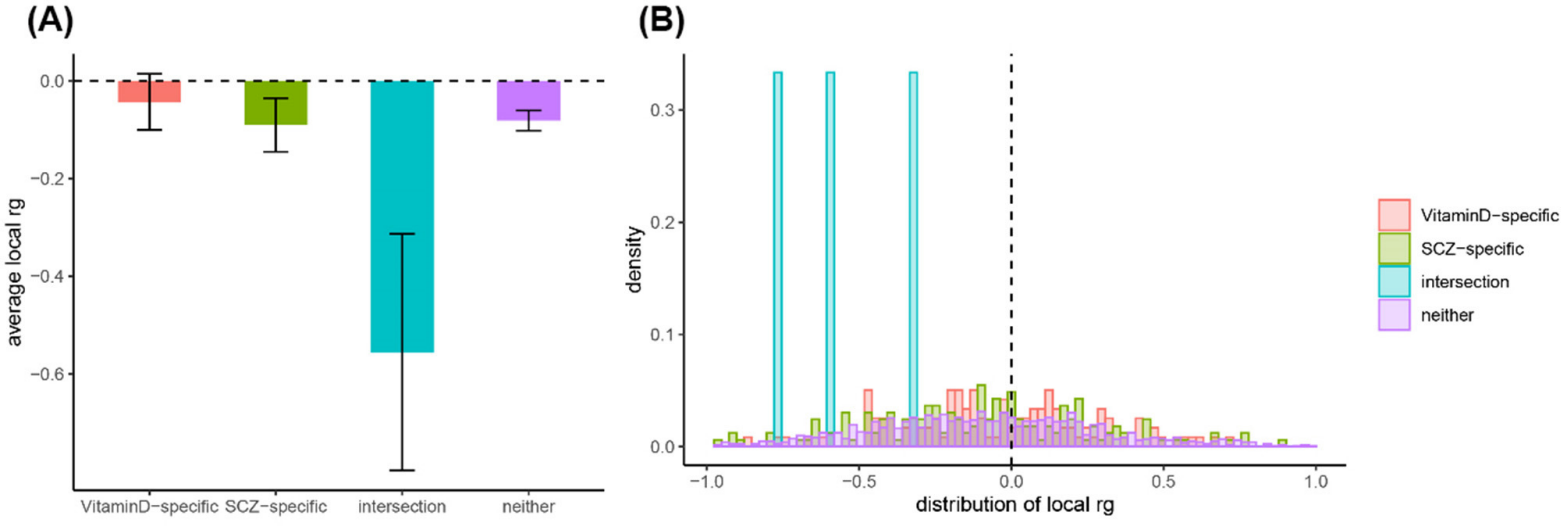
Analysis of local genetic correlation and heritability distribution across different genomic regions. **(A)** Average local genetic correlations (rg) for Vitamin D-specific, SCZ-specific, and interaction regions, highlighting the significant negative correlation in the interaction regions. **(B)** Frequency distribution of local genetic correlations, showing a concentration of negative correlations in interaction regions, suggesting a complex negative genetic relationship between Vitamin D and SCZ in these areas.

In this study, we observed a significant genetic correlation between 25OHD and SCZ (rg = −0.083, *P* = 1 × 10^−4^) by LDSC. The intercept was −0.008 (with a standard error of 0.007), excluding the possibility of sample overlap between 25OHD and SCZ data. The LDSC method without constraining the intercept yielded consistent results (rg = −0.091, *P* = 3.48 × 10^−9^). Additionally, the HDL method also provided nearly identical results (rg = −0.103, *P* = 2.96 × 10^−4^). Supplementary Table S1 provides a detailed breakdown of the genetic correlations between 25OHD and SCZ, showing the consistency of results across different methods such as LDSC and HDL. The table highlights the significant negative genetic correlation observed and further supports the robustness of these findings. By using HESS, we estimated significant local genetic correlations in some genomic regions and observed negative genetic overlaps in these overlapping regions. But no significant difference was observed in respective local genetic heritability estimated for SCZ and 25OHD (Figure 2 and Supplementary Table S2).

### 3.2 Pleiotropic GeneLoci identified for 25OHD and SCZ

**Figure 3:**
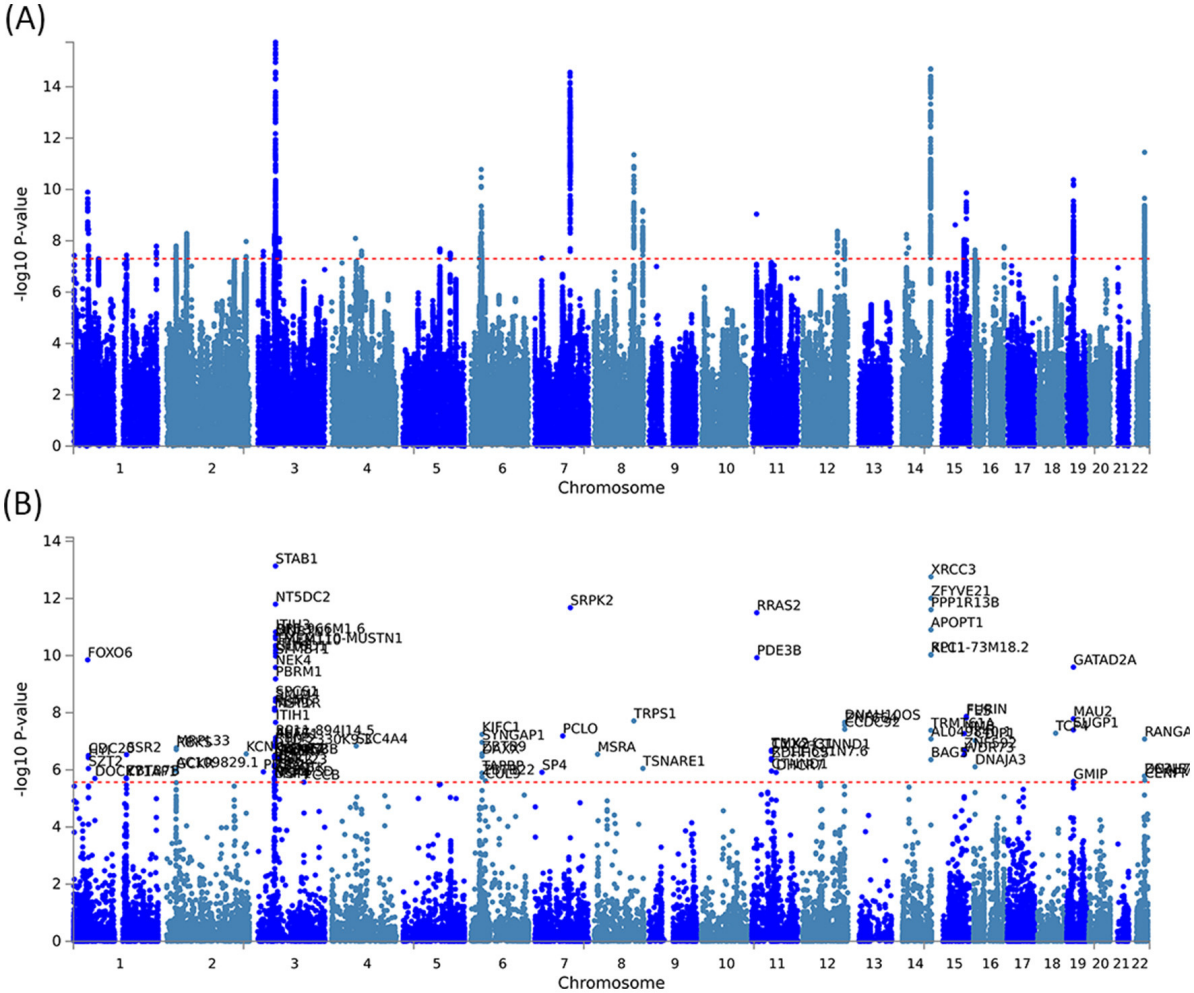
Manhattan plots illustrating pleiotropic associations between 25OHD and SCZ. **(A)** Manhattan plot showing pleiotropic SNP signals between 25OHD and SCZ, with significant associations observed on chromosomes 3, 7, 8, 14, and 19. **(B)** Manhattan plot highlighting pleiotropic genes identified by the MAGMA method, such as STAB1 and NT5DC2, which are significantly associated with both 25OHD and SCZ.

**Figure 4:**
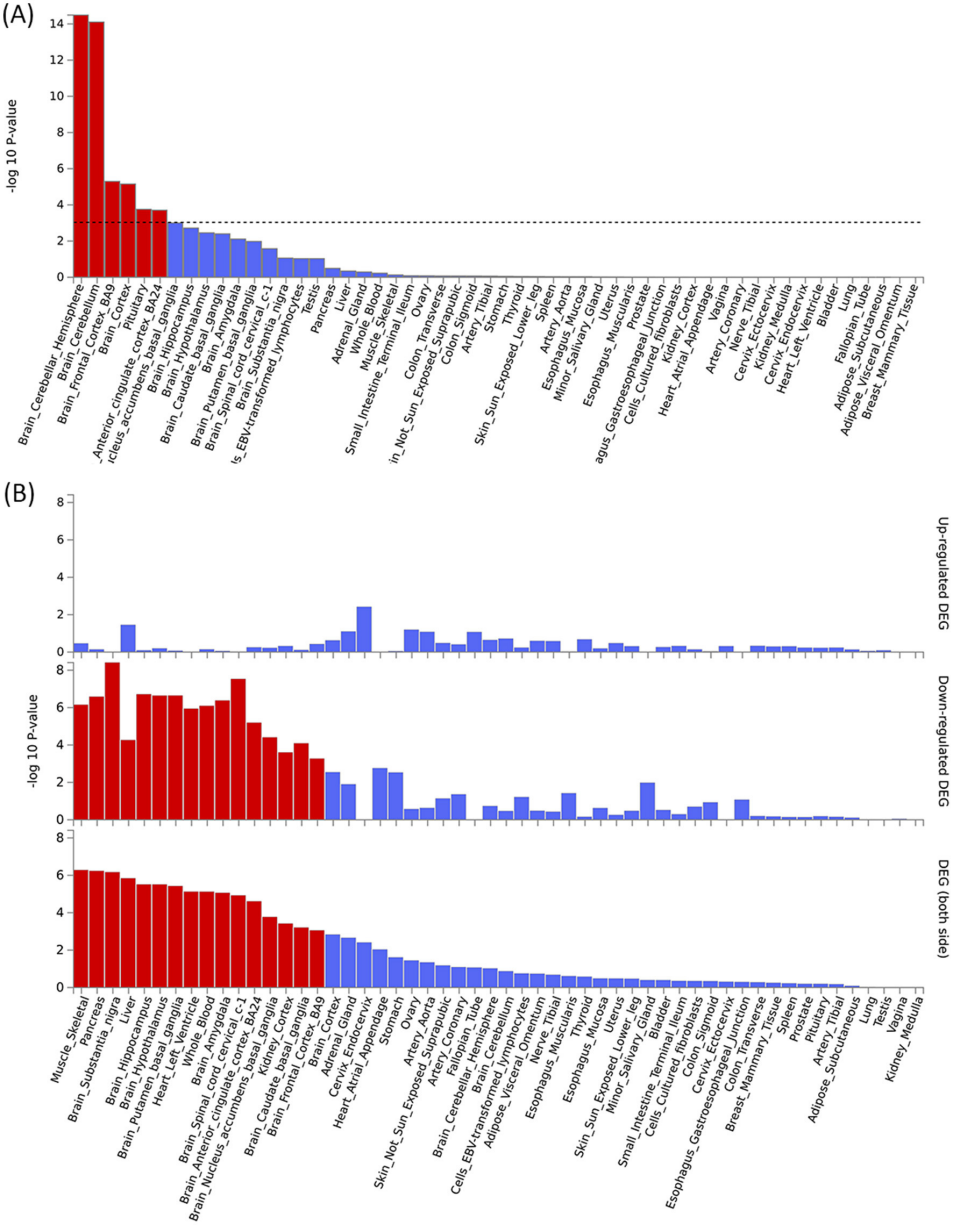
Tissue-specific analysis and gene enrichment of pleiotropic signals between 25OHD and SCZ. **(A)** Tissue-specific analysis reveals significant enrichment of pleiotropic signals in brain regions such as the cortex, frontal cortex, and cerebellum. **(B)** The enrichment of pleiotropic MAGMA genes is particularly pronounced in brain tissues, with both upregulated and downregulated genes showing significant associations, highlighting the central role of these regions in the genetic relationship between 25OHD and SCZ.

By using PLACO analysis, we identified a total of 35 independent pleiotropic loci between 25OHD and SCZ, with eight genomic regions (FOXO6, GCKR, NEK4, CTB-35F21.1, RP11-328J6.1, PPP1R13B, CDIP1, and FANCA) having PP.H4 values greater than 0.7, indicating their significance in the association between 25OHD and SCZ. Figure 3A is the Manhattan plot of the identified signals, and detailed information on the identified pleiotropic loci was presented in Table 1 and Supplementary Table S3, including the genomic positions and their associated genes. We didn't find any evidence of genome inflation in the QQ Plot (Supplementary Figure S1), indicating that the results are free from systematic biases. Additionally, Supplementary Figure S2 provides essential information on each genomic risk locus, offering a detailed overview of the genetic architecture and variability across loci in the study. Supplementary Figure S3 shows significant enrichment of pleiotropic SNPs in intronic and intergenic regions, particularly in introns, suggesting a key role for non-coding regions in the genetic overlap between 25OHD and SCZ. Other regions, such as downstream and exonic areas, also show some enrichment but to a lesser extent. Regional plots for each risk locus are presented in Supplementary Figures S4–S11. Gene set enrichment analysis using MAGMA was performed based on the results of pleiotropy (Table 2), and the top 3 pathways were statins inhibit cholesterol production, plasma lipoprotein remodeling in responder group and vitamin D Metabolism. Furthermore, we identified nominal significant enrichment in the top six tissues (*P* < 1 × 10^−3^) using MAGMA tissue-specific analysis (Figure 4A), with five of these significantly enriched tissues being of cerebral origin, including the cerebellar hemisphere, cerebellum, and frontal cortex (BA9). These brain regions exhibited the highest enrichment levels, underscoring their potential involvement in the genetic relationship between 25OHD and SCZ (Supplementary Table S4). Additionally, the pituitary gland was also among the significantly enriched tissues, further highlighting the role of brain-related tissues in this association. It is worth noting that this section of the MAGMA gene set and tissue-specific analysis was conducted using the complete distribution of SNP *p*-values.

**Table 1:** Information on 35 pleiotropic loci identified between 25OHD and SCZ.

**Genomic locus**	**Locus region**	**Lead SNP**	**P**	**P_adj_**	**Symbol**	**PP.H4**
1p36.32	1:2274325-2631294	rs6688934	3.74E-08	0.037	PLCH2	0.024
1p34.2	1:41467273-41933422	rs10218712	1.28E-10	< 0.001	FOXO6	0.986
1p34.2	1:43760070-44484269	rs11210887	3.63E-09	0.004	PTPRF	0.014
1q22	1:155043400-156081240	rs12043212	3.65E-08	0.037	RP11-336K24.5	0.606
1q43	1:243019418-244110411	rs12743378	1.63E-08	0.016	SDCCAG8	0.661
2p23.3	2:27284987-28503941	rs6744393	1.60E-08	0.016	GCKR, AC109829.1	0.718
2p16.1	2:58132104-59120088	rs2215966	5.22E-09	0.005	LINC01122	0.581
2q37.1	2:233550713-233821491	rs6704768	1.07E-08	0.011	GIGYF2	0.017
3p24.3	3:16687078-17151806	rs9876137	2.61E-08	0.026	PLCL2	0.037
3p21.31	3:49044713-50593986	rs6765484	5.81E-09	0.006	RBM6	0.676
3p21.1	3:52214640-53539241	rs7646741	1.84E-16	< 0.001	NEK4	0.840
3p14.1	3:63674722-64107217	rs704364	8.14E-09	0.008	ATXN7	0.201
4q13.2	4:69497139-70517902	rs41297381	8.06E-09	0.008	UGT2B4	0.343
4q22.1	4:87983900-88430396	rs28445336	2.53E-08	0.025	HSD17B11	0.444
5q21.3	5:108642367-109222729	rs253245	2.08E-08	0.021	KRT18P42, AC012603.1	0.515
5q31.2	5:138346820-139391816	rs4912756	3.04E-08	0.030	CTB-35F21.1	0.738
6p22.2	6:25147518-27124904	rs198811	4.83E-08	0.048	HIST1H2AC	0.040
6p21.32	6:33191338-33864288	rs10807124	3.89E-08	0.039	SYNGAP1	0.388
7p15.3	7:21285713-21713646	rs6461561	4.73E-08	0.047	SP4	0.377
7q22.3	7:104410300-105096449	rs2428162	2.74E-15	< 0.001	LINC01004, RP11-325F22.2	0.151
8q23.3	8:116481788-117135574	rs1109537	4.47E-12	< 0.001	LINC00536	0.326
8q24.3	8:143250850-143534403	rs13262595	6.51E-10	0.001	TSNARE1	0.005
11p15.2	11:13674659-14268378	rs11023064	9.17E-10	0.001	SPON1	0.316
12q23.2	12:103261749-103906212	rs10860964	4.24E-09	0.004	RP11-328J6.1, C12orf42	0.947
12q24.31	12:124149975-124597359	rs79478560	1.02E-08	0.010	DNAH10	0.649
14q13.1	14:33257822-33359973	rs12883788	5.69E-09	0.006	AKAP6, NPAS3	0.061
14q21.1	14:38783868-40213851	rs8011075	1.86E-08	0.019	SEC23A	0.279
14q32.33	14:103748844-104622780	rs4906378	2.00E-15	< 0.001	PPP1R13B	0.805
15q21.3	15:58718230-58785679	rs12914626	2.41E-09	0.002	RP11-355N15.1	0.586
15q25.2	15:84263509-85747441	rs62019457	9.06E-09	0.009	UBE2Q2P1	0.029
15q26.1	15:91390454-91481048	rs6224	1.37E-10	< 0.001	FURIN	0.312
16p13.3	16:4375753-4811179	rs2058811	2.30E-08	0.023	CDIP1	0.966
16q24.3	16:88963011-90239983	rs78004870	1.71E-08	0.017	FANCA	0.987
19p13.11	19:19068718-20159711	rs2965185	4.23E-11	< 0.001	GATAD2A	0.000
22q13.2	22:40818166-42577604	rs9607782	3.57E-12	< 0.001	RP1-85F18.5	0.214

Lead SNP was the SNP with minimum *P* values within the corresponding locus. PP.H4 was the posterior probability of H4 calculated by coloc analysis; the Locus boundary was defined as “chromosome: start-end.” PP.H4, the posterior probability of H4; 25OHD, 25-hydroxy vitamin D; SCZ, schizophrenia.

**Table 2:** MAGMA gene-set analysis results (top 10).

**Gene set**	**N genes**	**Beta**	**SE**	***P***	***P_*adj*_***
WP_STATIN_INHIBITION_OF_CHOLESTEROL_PRODUCTION	29	0.798	0.188	1.14E-05	0.195
REACTOME_PLASMA_LIPOPROTEIN_REMODELING	32	0.728	0.182	3.05E-05	0.518
WP_VITAMIN_D_METABOLISM	9	1.442	0.367	4.33E-05	0.736
NIKOLSKY_BREAST_CANCER_16Q24_AMPLICON	52	0.947	0.242	4.47E-05	0.759
WP_METABOLIC_PATHWAY_OF_LDL_HDL_AND_TG_ INCLUDING_DISEASES	17	0.981	0.250	4.52E-05	0.768
GOBP_HIGH_DENSITY_LIPOPROTEIN_PARTICLE_REMODELING	15	1.084	0.278	4.91E-05	0.834
GOCC_POSTSYNAPTIC_SPECIALIZATION	339	0.218	0.056	4.97E-05	0.845
GOBP_REGULATION_OF_FEAR_RESPONSE	8	1.386	0.362	6.32E-05	1.000
KEGG_DRUG_METABOLISM_OTHER_ENZYMES	47	0.603	0.159	7.12E-05	1.000
GOBP_LIPOPROTEIN_BIOSYNTHETIC_PROCESS	94	0.367	0.097	8.23E-05	1.000

25OHD, 25-hydroxy vitamin D; SCZ, schizophrenia; MAGMA, multivariate analysis of genomic annotation. P_adj_ was estimated by Bonferroni method.

### 3.3 Identification of priority pleiotropic genes and functional enrichment

The pleiotropic genes were mapped using three different approaches, including the mapping of nearby genes based on the physical position of lead SNPs, MAGMA gene-level associations, and eQTL analysis (Supplementary Table S5). Supplementary Figure S12 and Supplementary Table S6 provide a comprehensive heatmap of gene expression levels across various tissues, with notable expression observed in the brain cortex, hippocampus, and cerebellum. Supplementary Figure S13 and Supplementary Table S7 illustrate the enrichment patterns of gene expression across multiple tissues. Significant enrichment is observed in regions such as the brain cortex, frontal cortex, and hippocampus. These findings suggest that these genes may have important roles in neurological functions, potentially linked to the genetic relationship between 25OHD and SCZ. MAGMA gene analysis identified a total of 105 pleiotropic genes (*P* < 2.69 × 10^−6^ = 0.05/18,565) (Figure 3B and Supplementary Table S8). Supplementary Figure S14 presents the QQ plot, showing a clear deviation from the expected line, particularly in the upper tail. This suggests the presence of significant genetic associations that contribute to the relationship between 25OHD and SCZ. The expression values of these genes across 54 different tissues can be found in Supplementary Figure S15 and Supplementary Table S9. Many genes were observed to be differentially expressed across the 13 brain tissues (e.g., STAB1, GLYCTK, and FOXO6), while several genes (e.g., CDC20, CENPM, and KIFC1) exhibited high expression in EBV-transformed lymphocytes, testis, liver, and whole blood. Tissue-specific enrichment analysis revealed the enrichment of these pleiotropic genes in skeletal muscle and various brain tissues (Supplementary Table S10 and Figure 4B). We further conducted pathway analysis (KEGG, Wiki, GO) after integrating the gene mapping based on positional information and MAGMA gene analysis (Supplementary Figure S16). We observed that these genes were significantly enriched in pathways such as 15q25 copy number variation, presynaptic active zone cytoplasmic component, and hyaluronan metabolic process. Through cell-specific enrichment analysis, the enrichment was primarily observed in three cell phenotypes: “Descartes fetal spleen afp alb positive cells”, “Lake adult kidney c3 proximal tubule epithelial cells s1 s2” and “Fan embryonic ctx nsc 2” (Supplementary Figure S17). eQTL mapping analysis was further conducted based on 13 brain and whole blood tissues, identifying a total of 329 pleiotropic genes. For detailed information, please refer to Supplementary Table S5. The overlap of genes between different methods is illustrated in Supplementary Figure S18 and a total of 11 (28%) pleiotropic genes were identified in all three mapping approaches (Supplementary Table S11).

### 3.4 Immune-related mechanisms shared between 25OHD and SCZ

**Figure 5:**
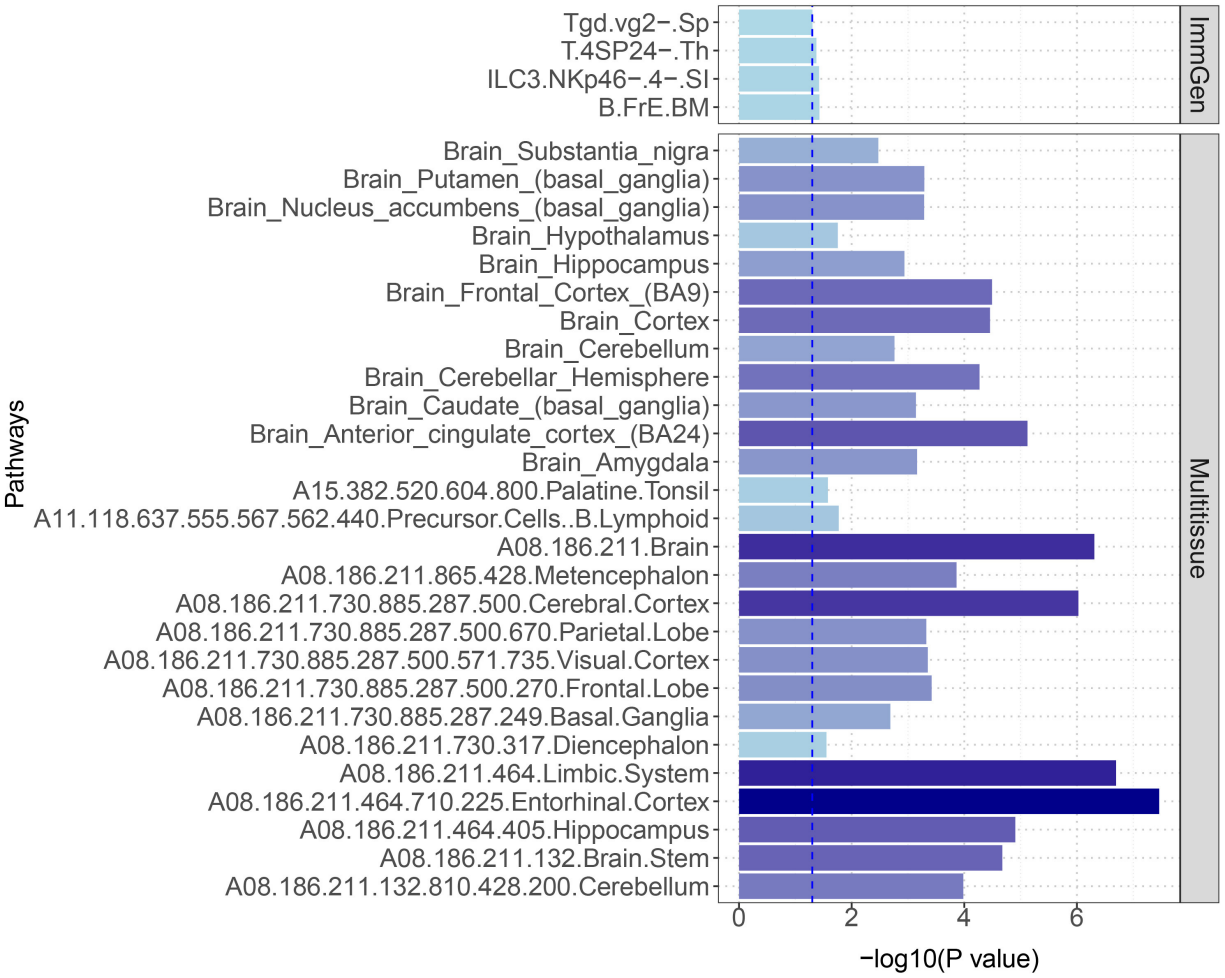
S-LDSC estimates of heritability enrichment across different immune cells and brain tissues. The figure highlights significant heritability enrichment in specific immune cells, such as Tgd.vg2-.Sp and T.4SP24-.Th, as well as in key brain regions including the entorhinal cortex, limbic system, and cerebral cortex. These findings suggest critical roles for both immune cells and these specific brain regions in the genetic relationship between 25OHD and SCZ.

Through S-LDSC, we observed significant enrichment of SNP heritability in 27 brain tissues and four immune cell phenotypes, which was significantly associated with the pleiotropic results between 25OHD and SCZ (*P* < 0.05) (Figure 5 and Supplementary Table S12). When analyzing the enrichment of immune traits from ImmGen, we also observed two cell phenotypes enriched in the T-cell panel: Tgd.vg2-.Sp and T.4SP24-.Th. Furthermore, within the innate lymphocyte panel, ILC3.NKp46-0.4-.SI, and within the B-cell panel, B.FrE.BM, were identified, suggesting potential shared immune mechanisms.

### 3.5 Multi-trait colocalization analysis to pinpoint critical immune traits

Our multi-trait colocalization analysis, conducted using the HyPrColoc method, unveiled shared genetic loci among a diverse array of immune cell phenotypes, 25OHD and SCZ. Within the realm of immune cells, we observed significant genetic associations with several traits, such as CD80 on myeloid DCs, CD80 on CD62L+ myeloid DCs, and CD28 on CD39+ activated Treg cells (Supplementary Table S13). Notably, these findings included traits from different panels like cDC, Treg, B cells, and TBNK, which play pivotal roles in the immune system.

### 3.6 Causal association between 25OHD and SCZ

We employed a bidirectional two-sample MR approach to infer causality between exposure and outcome. The results provided support for a significant causal effect of SCZ on 25OHD levels, but failed to reveal a causal relationship from 25OHD to SCZ (Table 3). We employed both the global test of MR-PRESSO and the intercept term of MR-Egger to detect variants with disproportional effects and potential directional pleiotropy. Additionally, we utilized scatter plots and funnel plots to evaluate the stability of the association (Supplementary Figure S19). The scatter plot revealed the absence of multi-effect outliers that could influence causal inference. Meanwhile, the funnel plot displayed a balanced distribution of causal estimates around the effect estimates, confirming the robustness of the results. Supplementary Table S14 presents the information for the IVs.

**Table 3:** Bidirectional Mendelian randomization analysis results of 25OHD and SCZ.

**Exposure**	**Outcome**	**Methods**	**Estimate (95%CI)**	***P***	**Heterogeneity test**	
					**Q**	***P***
25OHD	SCZ	IVW (fixed)	0.968 (0.904, 1.037)	0.358	171.493	< 0.001
		IVW (random)	0.968 (0.889, 1.055)	0.461		
		MR-Egger (slope)	0.95 (0.846, 1.068)	0.389		
		MR-Egger (intercept)	0.001 (−0.003, 0.004)	0.634		
		Weighted mode	0.946 (0.875, 1.024)	0.169		
		Weighted median	0.933 (0.837, 1.04)	0.212		
		DIVW	0.968 (0.886, 1.058)	0.474		
		MR-RAPS	0.949 (0.868, 1.038)	0.254		
SCZ	25OHD	IVW (fixed)	0.983 (0.978, 0.988)	4.35E-10	392.902	< 0.001
		IVW (random)	0.983 (0.974, 0.992)	1.58E-04		
		MR-Egger (slope)	0.973 (0.939, 1.008)	0.126		
		MR-Egger (intercept)	0.001 (−0.002, 0.003)	0.554		
		Weighted mode	0.973 (0.955, 0.991)	0.003		
		Weighted median	0.983 (0.974, 0.992)	2.83E-04		
		DIVW	0.983 (0.974, 0.991)	1.40E-04		
		MR-RAPS	0.984 (0.975, 0.993)	5.60E-04		

MR, Mendelian Randomization; IVW, inverse variance weighted; DIVW, debiased IVW; CI, confidence interval; 25OHD, 25-hydroxy vitamin D; SCZ, schizophrenia.

## 4 Discussion

This study, based on extensive sample data from GWAS research, employed the LDSC method and identified a pronounced genetic correlation between 25OHD and SCZ (rg = −0.083, *P* = 1 × 10^−4^). S-LDSC extended this by assessing whether specific tissues, displayed significant enrichment of heritability, helping to localize the genetic influence to biologically relevant tissues. Using HESS, we estimated local genetic correlations, which allowed us to identify specific genomic regions where overlapping genetic effects are concentrated. Pleiotropy analysis then identified pleiotropic loci and found notable gene enrichment in brain tissues, suggesting a potential shared biological basis. To examine immune-related effects, HyPrColoc analysis integrated immune cell data to uncover shared genetic information across 25OHD, immune traits, and SCZ. Finally, bidirectional MR was used to explore potential causal effects between SCZ and 25OHD, allowing us to infer the directionality of their association.

Our study confirmed a significant genetic correlation between 25OHD and SCZ. Bidirectional two-sample MR analysis revealed that SCZ is a risk factor for 25OHD deficiency. Worldwide, the prevalence of 25OHD deficiency is as high as 37.6% in newborns and the elderly (48). A meta-analysis of 25OHD deficiency prevalence revealed that the overall prevalence of 25OHD deficiency in SCZ patients is 65.3%. Individuals with 25OHD deficiency are 2.16 times more likely to develop SCZ compared to those with sufficient levels of 25OHD (24). A more recent meta-analysis found that this prevalence has increased to 70% (23). Two studies based on Danish biobanks have confirmed the association between neonatal 25OHD deficiency and an increased risk of SCZ (21, 22). There is evidence to suggest that genetic variations associated with psychiatric disorders, including major depressive disorder (MDD), bipolar disorder (BIP), and SCZ, are related to lower concentrations of 25OHD. People with risk allele genes associated with psychiatric disorders may have lower 25OHD levels, regardless of clinical diagnosis (49). Most evidence suggests an association between 25OHD deficiency and cardiovascular diseases (CVD) (50). A recent randomized controlled trial involving 21,315 participants found a lower incidence of major cardiovascular events in the group receiving vitamin D3 supplementation (60,000 IU/month) compared to the placebo group, suggesting that vitamin D supplementation may reduce the occurrence of major CVD events (51). Our research findings suggest that individuals with a family history of SCZ should be vigilant about 25OHD deficiency to prevent the later development of CVD events.

Furthermore, a PLACO pleiotropy analysis of both disorders revealed 35 pleiotropic loci, with eight genomic regions (FOXO6, GCKR, NEK4, CTB-35F21.1, RP11-328J6.1, PPP1R13B, CDIP1, and FANCA) having PP.H4 values >0.7, indicating their significance in the association between 25OHD and SCZ. NEK4 has been reported as a shared genetic signal between BIP and SCZ (52). The study identified NEK4 as one of the risk genes associated with neuropsychiatric and substance use disorders. Pathway enrichment analysis revealed an enrichment of XWAS signals in 25OHD gene sets. This suggests that NEK4 may play a significant role in vitamin D metabolic pathways, potentially influencing the risk of disorders such as SCZ. A study identified that alternative splicing of NEK4 is regulated by sQTLs, which are significantly enriched in schizophrenia-associated loci. This suggests that dysregulation of NEK4 splicing may be a key genetic mechanism contributing to SCZ risk (53). A Mendelian randomization study identified a total of 31 promising drug targets for psychiatric disorders, with NEK4 being one of the significant genes specifically associated with SCZ (54). A summary data-based Mendelian randomization (SMR) analysis found that risk alleles in the chromosome 3p21.1 region are associated with NEK4 mRNA expression, and these alleles are significantly linked to SCZ (55). Another study involving 133 first-episode SCZ patients observed a significant association between the severity of SCZ negative symptoms and the risk gene FOXO6, which is associated with BIP (56). A study identifies FOXO6 as a novel candidate gene for SCZ through its pleiotropic effects on educational attainment (EA) and SCZ, revealed by using EA as a proxy phenotype (57). GCKR has been identified as one of the genes associated with alcohol use disorders. Moreover, it exhibits a positive genetic correlation with SCZ, indicating a potential connection between GCKR and SCZ within the framework of alcohol use disorders (58). In summary, among the 35 pleiotropic loci identified, NEK4 shows the strongest evidence and is considered a potential target for the association between 25OHD and SCZ. While there are some studies reporting the relationship between FOXO6 and GCKR with SCZ, there are no reported associations with 25OHD, warranting further investigation.

Tissue-specific analysis revealed significant enrichment of these pleiotropic genes in six tissues, with five originating from brain tissues (*P* < 1 × 10^−3^). Our multi-effect results were further subjected to gene analysis using MAGMA, identifying 105 pleiotropic genes. Enrichment analysis demonstrated that these genes are enriched in skeletal muscle and various brain tissues. This emphasizes the potential roles of these gene loci in brain-related processes and their functional impact on skeletal muscle. We further conducted pathway enrichment analysis and found significant enrichment in five pathways: “15q25 copy number variation,” “Presynaptic active zone cytoplasmic component,” “Hyaluronan metabolic process,” “Glyoxylate and dicarboxylate metabolism,” and “Oxidoreductase activity, acting on a sulfur group of donors.” In a large-scale GWAS meta-analysis investigating the genetic determinants of smoking quantity and their relevance to SCZ, multiple loci were identified. Notably, among these loci, the 15q25 region emerged as a common genetic locus associated with these traits. This locus has been linked to alterations in CHRNA5 expression in the brain (59). Perineuronal nets (PNNs) are highly organized mesh-like networks composed of extracellular matrix molecules surrounding neuronal cell bodies and proximal dendrites. The main components of PNNs include hyaluronan synthase, cartilage link protein-1, and chondroitin sulfate proteoglycans (60). It has been reported that PNN deficiencies can lead to frontal cortex dysfunction in individuals with SCZ, and abnormal PNN formation may increase the risk of SCZ episodes (61). In the context of liver fibrosis induced by sodium arsenite, 25OHD intervention using calcitriol significantly reduces hyaluronic acid levels, suggesting a potential protective effect of vitamin D on liver fibrosis by modulating hyaluronan metabolism (62). 25OHD deficiency may lead to reduced PNN function, resulting in abnormal cortical gamma band oscillations, thereby increasing the risk of SCZ and worsening the cognitive symptoms of SCZ (63). These observations indicate that the hyaluronan metabolism pathway could be a crucial link between 25OHD and the pathophysiology of SCZ. The synthesis and activation of vitamin D involve multiple enzymes, with some of these enzymes participating in redox reactions involving sulfur atoms within the organism. They alter the structure of vitamin D by introducing oxygen atoms into the molecule, thereby activating it (64). In conclusion, among the five significantly enriched pathways identified, “Hyaluronan metabolic process” shows the strongest evidence, indicating its potential role as a pathway linking 25OHD and SCZ. “15q25 copy number variation” and “Oxidoreductase activity” have some reported associations with SCZ or 25OHD, suggesting they are worthy of further investigation.

25OHD possesses potential immunomodulatory effects (65). Some epidemiological evidence suggests a significant association between 25OHD deficiency and an increased risk or exacerbation of infectious diseases and autoimmune conditions (66–68). A MR study has demonstrated a significant association between various immune cell phenotypes and the risk of SCZ (69). Many immune-related genes and pathways have been shown to be involved in neural development and neuronal function (70). Studies suggest that elevated neutrophil counts and C-reactive protein levels may be more strongly associated with the severity of SCZ (71). Through multi-trait colocalization analysis of GWAS data from 731 immune cell phenotypes, we conducted an in-depth investigation and observed shared genetic loci between multiple immune traits and both 25OHD deficiency and SCZ. We identified 19 immune cell phenotypes with significant genetic overlap (PP > 0.5), including cDC, Treg, B cell, and TBNK panels, among others. These molecules play critical roles in the immune system, deepening our genetic understanding of SCZ and underscoring the importance of the immune system in the disease's pathogenesis.

25OHD has properties that promote differentiation, inhibit proliferation, and prevent apoptosis in brain cells. Offspring of mice with 25OHD deficiency have been found to exhibit increased cell proliferation in the brain (72). Cell proliferation is indeed associated with brain structural abnormalities, and 25OHD deficiency has been shown to reduce the expression of neurotrophic factors such as nerve growth factor (NGF), glial cell line-derived neurotrophic factor (GDNF), and brain-derived neurotrophic factor in the brains of neonatal rats. This reduction could potentially lead to early brain dysfunction (73). Although MR results suggest a unidirectional effect of SCZ on 25OHD deficiency, this does not eliminate the possibility of a bidirectional relationship between these two factors. Given the intricate network of genetic correlations and pleiotropy, it is likely that the relationship between 25OHD and SCZ is multifaceted. One plausible explanation for this could be the complexity of SCZ etiology, which involves multiple genetic and environmental factors. SCZ pathogenesis is influenced by a wide range of environmental and lifestyle factors, including sunlight exposure, diet, and other socio-environmental elements, which play essential roles in vitamin D synthesis and metabolism (74–76). However, the potential positive effects of these factors on SCZ risk remain speculative (77). These factors add layers of complexity to SCZ etiology, making it unlikely that a single nutrient or metabolic factor could fully account for the disorder's development. In addition, vitamin D levels may play a more indirect or compensatory role rather than a primary causal role in SCZ. While our analysis found no direct causal link between 25OHD and SCZ, the inverse relationship where SCZ appears to influence 25OHD levels suggests that psychiatric conditions, lifestyle factors, or medication use in SCZ patients could negatively affect vitamin D metabolism or absorption. For instance, individuals with SCZ often have limited outdoor exposure and may exhibit poor dietary habits, both of which can lead to vitamin D deficiency (8, 78). Furthermore, antipsychotic medications, frequently prescribed for SCZ, have been shown to alter metabolic pathways, potentially affecting vitamin D levels (8). Several clinical randomized controlled trials have examined whether supplemental 25OHD levels improve symptoms of SCZ, but the results have been inconsistent. One study showed that vitamin D supplementation (50,000 IU vitamin D per week for 12 weeks) improved positive or negative symptoms of SCZ (79). Conversely, another study found no improvement in symptoms with vitamin D supplementation in patients using antipsychotic medications (80). Other small open-label studies have also shown inconsistent results (81, 82). The inconsistency of these trials may be attributed to varying baseline 25OHD levels, differences in patient profiles, or the impact of vitamin D on drug metabolism. Evidence suggests that vitamin D has a direct effect on drug metabolism by inducing CYP3A4, which may reduce the efficacy of antipsychotic medications that are CYP3A4 substrates (83). Studies have shown a negative correlation between serum vitamin D levels and the concentrations of such antipsychotics, indicating that while vitamin D may contribute to symptom relief (83), this effect could be counteracted by its impact on medication levels. Thus, while vitamin D supplementation remains important for overall health, particularly for those at risk of deficiency, current findings do not support its use as a primary or standalone treatment for SCZ. Instead, comprehensive approaches that consider the multifactorial nature of SCZ—including genetic, environmental, and lifestyle factors—are essential. For individuals at high risk of SCZ, strategies such as maintaining sufficient vitamin D levels through diet and sunlight exposure may offer general health benefits (9, 77), although their potential role in specifically reducing SCZ risk remains uncertain and requires further investigation. These lifestyle adjustments can help ensure adequate vitamin D, which may indirectly support health without the complexities introduced by direct supplementation in combination with antipsychotic treatment (84).

## 5 Limitation

This study has several limitations that should be considered. First, due to the use of summary-level data, we were unable to perform stratified analyses by key factors such as sex, age, or disease subtypes. While sex-specific effects were not explored, age-related differences and variations across SCZ subtypes could also influence the association between serum 25OHD levels and SCZ risk. Future studies utilizing individual-level data are necessary to assess potential interactions across these factors. Second, our analysis assumed a linear relationship between 25OHD and SCZ, as current MR methodologies primarily focus on linear associations. Although non-linear MR methods are emerging, they are still in their early stages and have significant limitations, particularly when applied to summary-level data. As a result, we were unable to robustly assess non-linear effects, which may provide deeper insights into the 25OHD-SCZ relationship. Methodological advancements and access to individual-level data could help address these issues in the future. Furthermore, the study predominantly included individuals of European ancestry, which may limit the generalizability of the findings to other ethnic populations. Genetic variations and environmental factors, such as differences in vitamin D metabolism, may vary across ethnic groups. We have noted that while several associations in MAGMA gene-set analysis were initially observed, they did not retain statistical significance after adjustment for multiple comparisons. We caution readers to interpret these findings conservatively, recognizing that the non-significant adjusted *p-*values may indicate that these associations are not robust to stringent statistical correction. Additionally, we have highlighted the need for further validation in independent cohorts to confirm the findings. Future research should include more diverse populations to enhance the external validity of the results. Lastly, while the MR approach strengthens causal inference, it is not without limitations. Our analysis may be subject to horizontal pleiotropy, where genetic variants influence SCZ risk through pathways independent of 25OHD. Although sensitivity analyses were performed to address this, residual pleiotropy cannot be entirely ruled out. Additionally, MR relies on the assumption that the genetic instruments are valid proxies for the exposure, which may not always hold true in complex traits like SCZ. Ultimately, the robustness of our results requires further validation through experimental and mechanistic studies to fully confirm the causal pathways.

## 6 Conclusions

Our study has unveiled the genetic correlation between 25OHD levels and SCZ, further supporting the hypothesis that 25OHD deficiency and SCZ have shared genetic mechanisms. We have identified pleiotropic genetic loci and pathways that link these two conditions. Importantly, our Mendelian randomization analysis suggests that SCZ influences 25OHD levels. This research may serve as a foundation for future investigations into shared genetic mechanisms and molecular interactions.

## Data Availability

The original contributions presented in the study are included in the article/Supplementary material, further inquiries can be directed to the corresponding authors.
