# Supplementary material for: Association between 25(OH) vitamin D and schizophrenia: shared genetic correlation, pleiotropy, and causality
Source: Front Nutr. 2024 Dec 13;11:1415132. doi: 10.3389/fnut.2024.1415132 (PMC11671254; doi:10.3389/fnut.2024.1415132)
Supplement: Supplementary file 2 [file Image_1.pdf]

Supplementary Material

Association between 25(OH) Vitamin D and Schizophrenia: Shared genetic Correlation, Pleiotropy and Causality

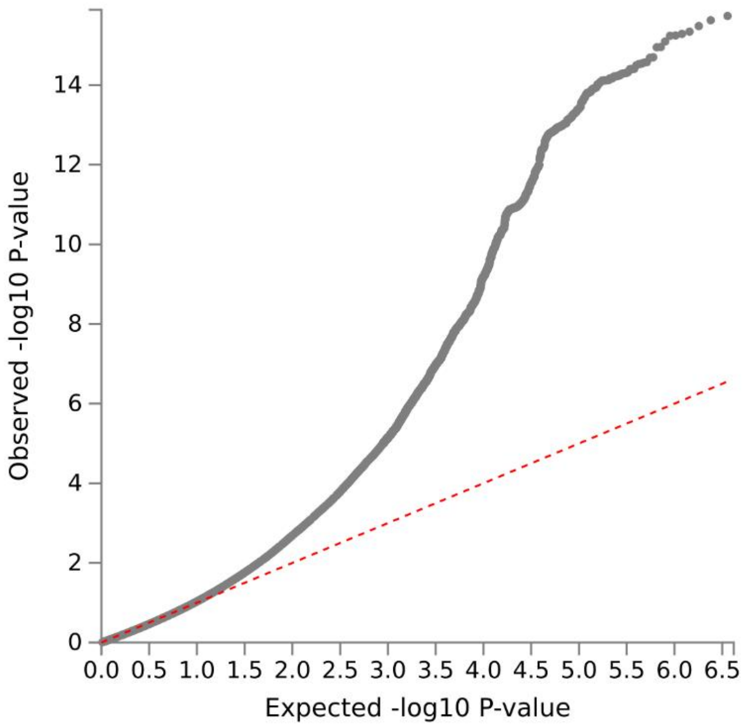

Figure S1. QQ plot of genome-wide PLACO.

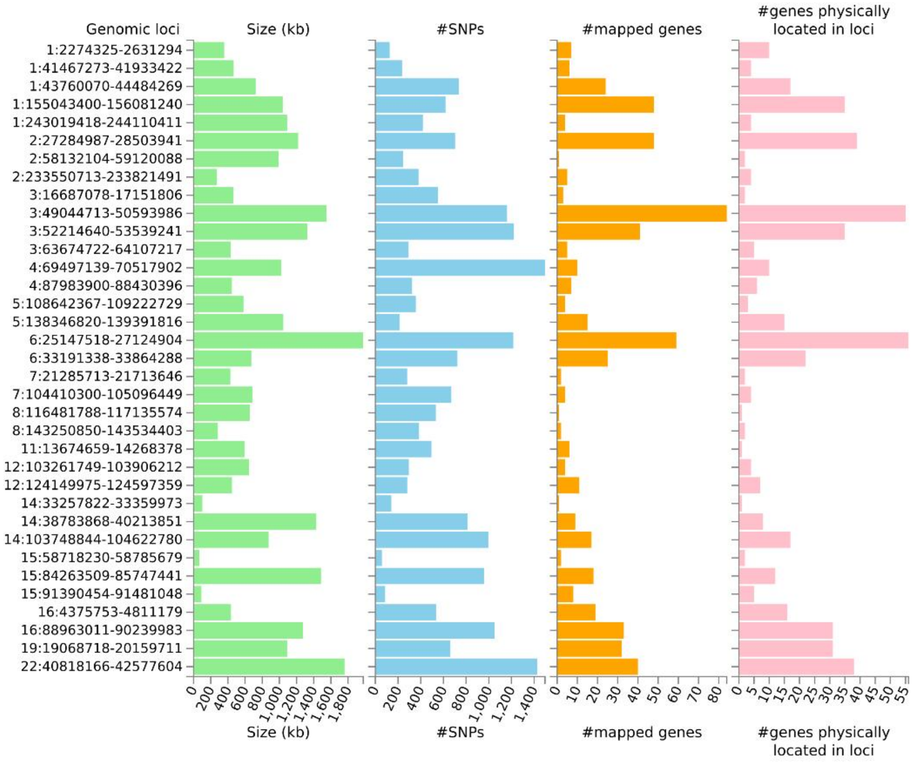

Figure S2. Summary of basic information for each genomic risk site.

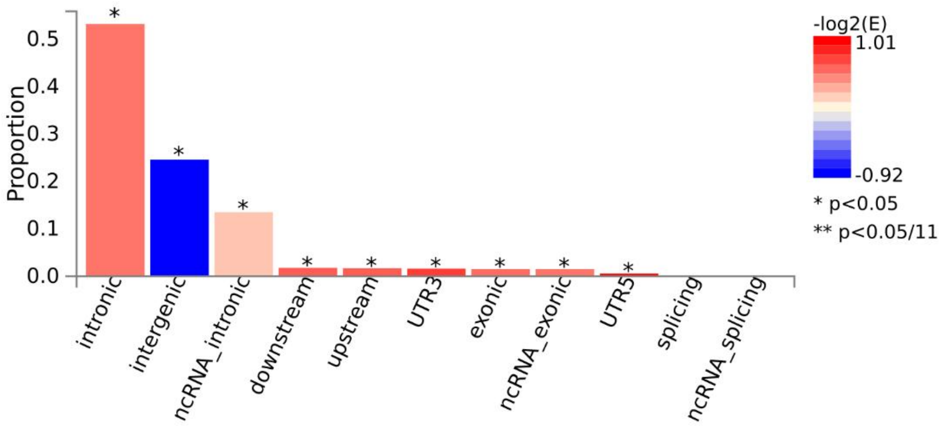

Figure S3. The functional impact of pleiotropic SNPs on genes.

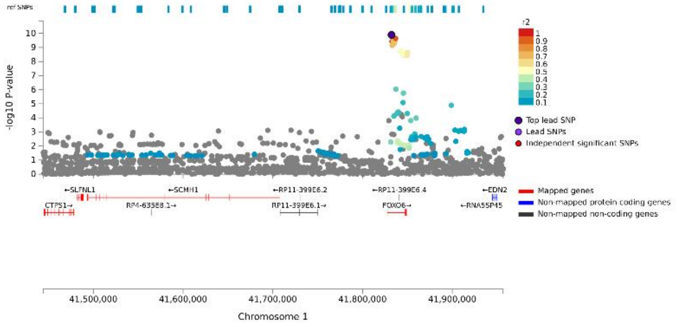

Figure S4. Regional plot of locus (1p34.2) (PP.H4 > 0.7).

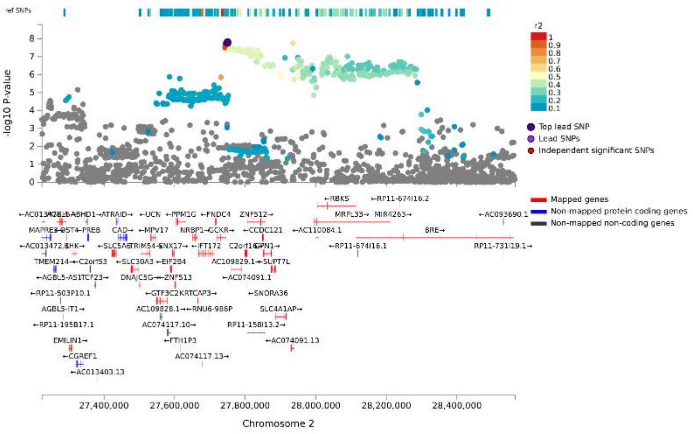

Figure S5. Regional plot of locus (2p23.3) (PP.H4 > 0.7).

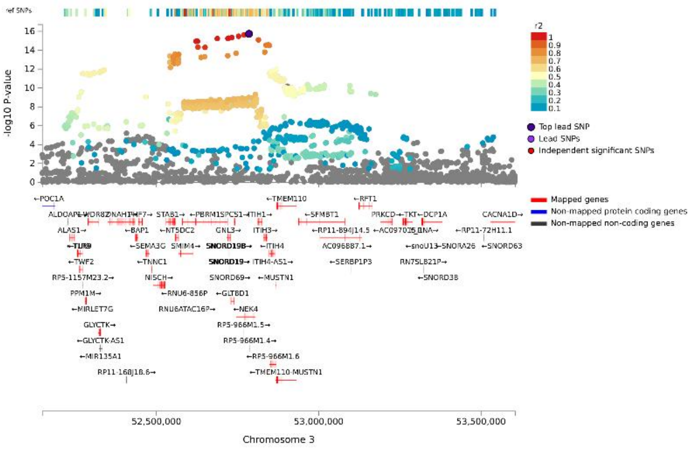

Figure S6. Regional plot of locus (3p21.1) (PP.H4>0.7).

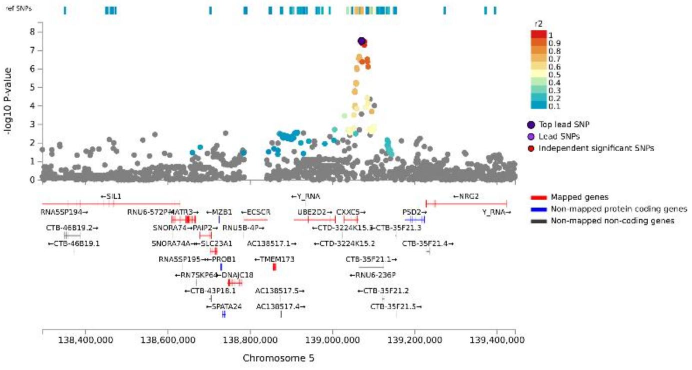

Figure S7. Regional plot of locus (5q31.2) (PP.H4 > 0.7).

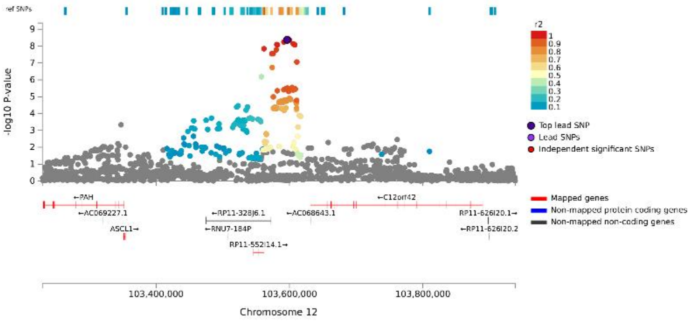

Figure S8. Regional plot of locus (12q23.2) (PP.H4 > 0.7).

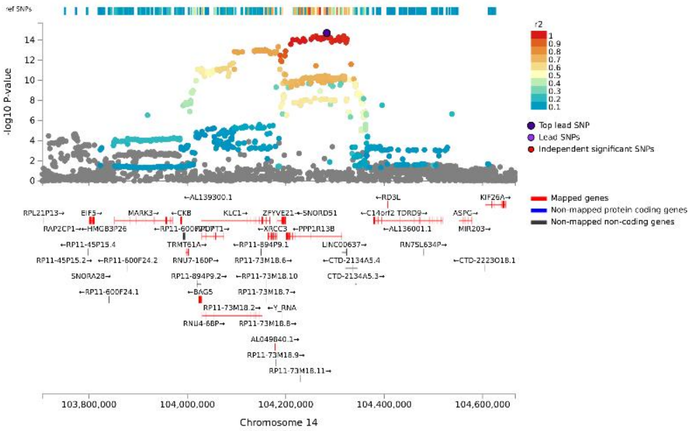

Figure S9. Regional plot of locus (14q32.33) (PP.H4 > 0.7).

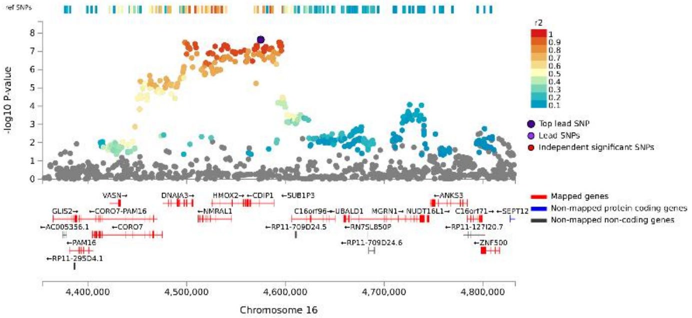

Figure S10. Regional plot of locus (16p13.3) (PP.H4 > 0.7).

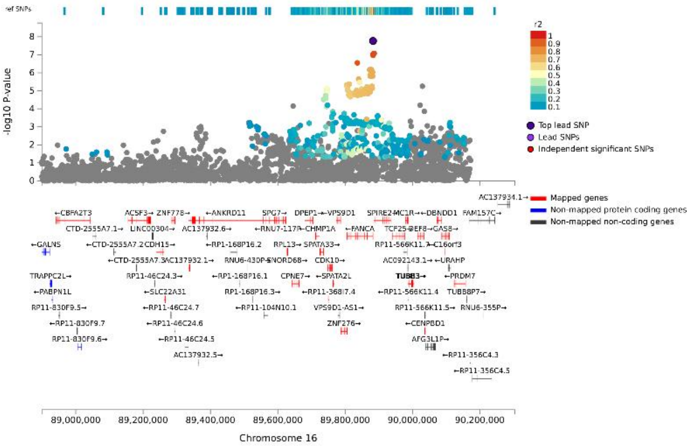

Figure S11. Regional plot of locus (16q24.3) (PP.H4 > 0.7).

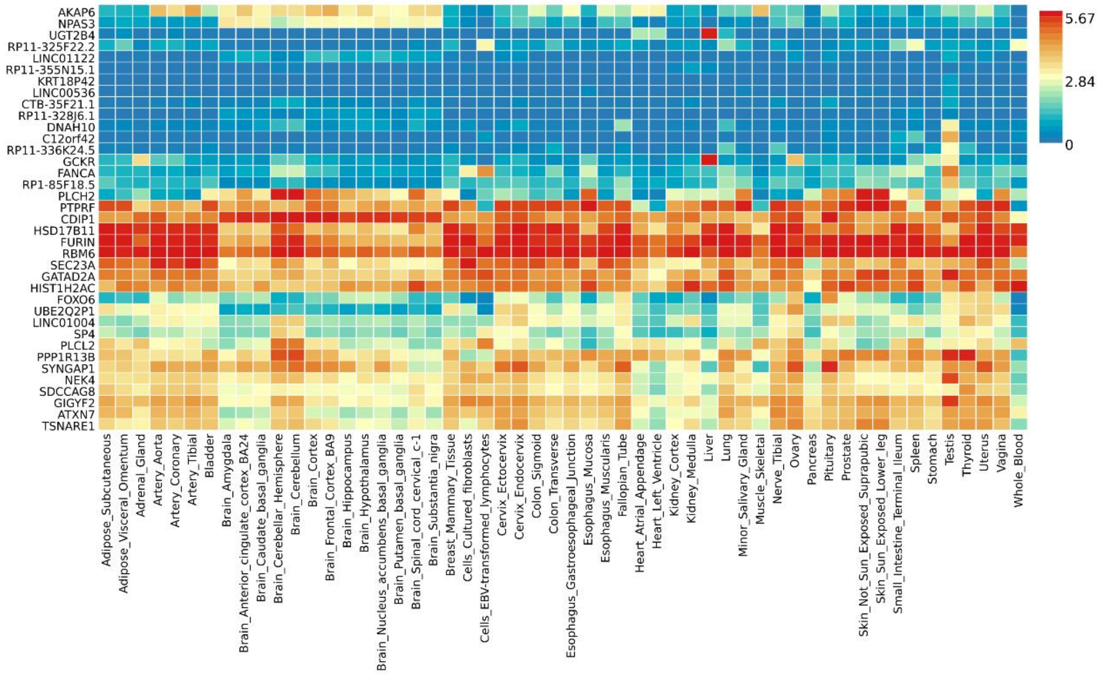

Figure S12. Expression of pleiotropic genes in different

tissues.

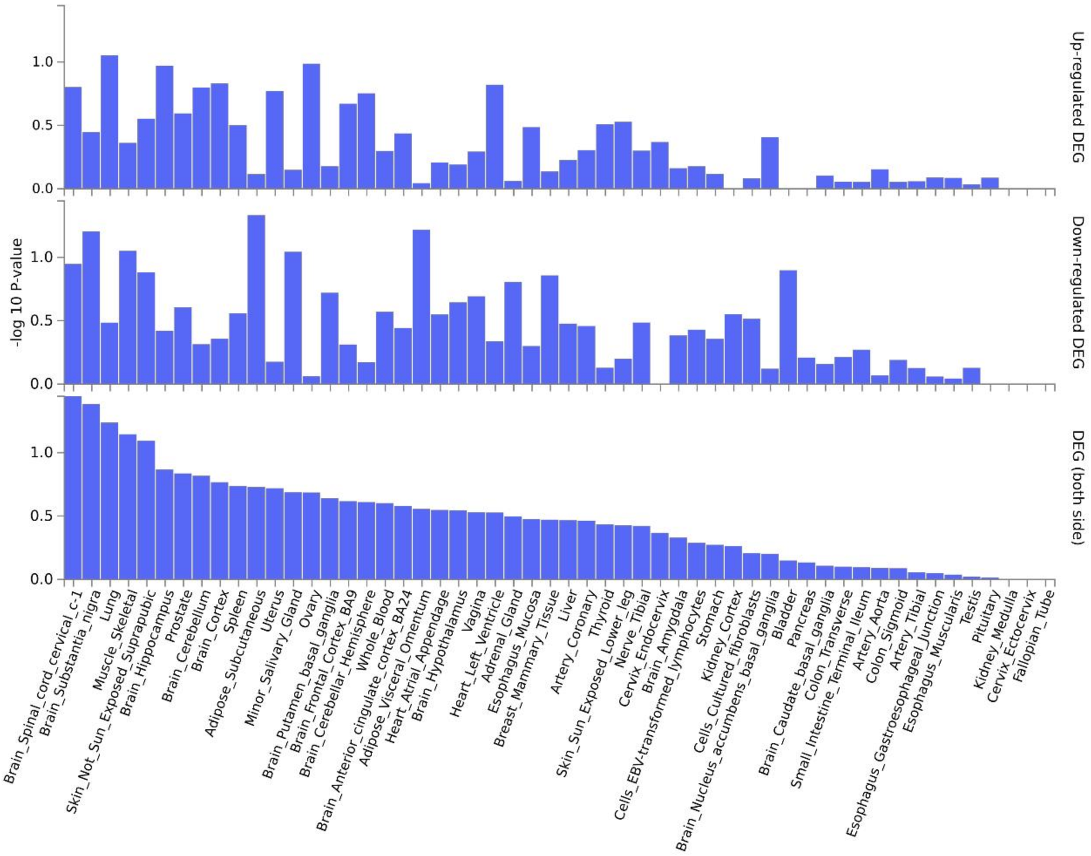

Figure S13. Enrichment of pleiotropic genes in different tissues.

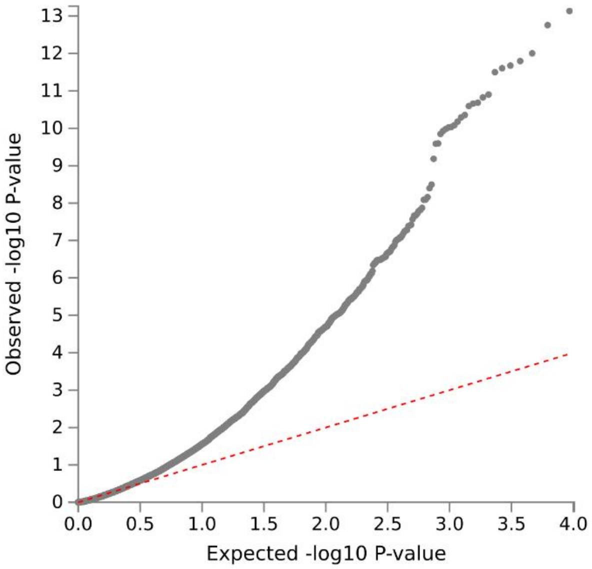

Figure S14. QQ plot of MAGMA gene test.

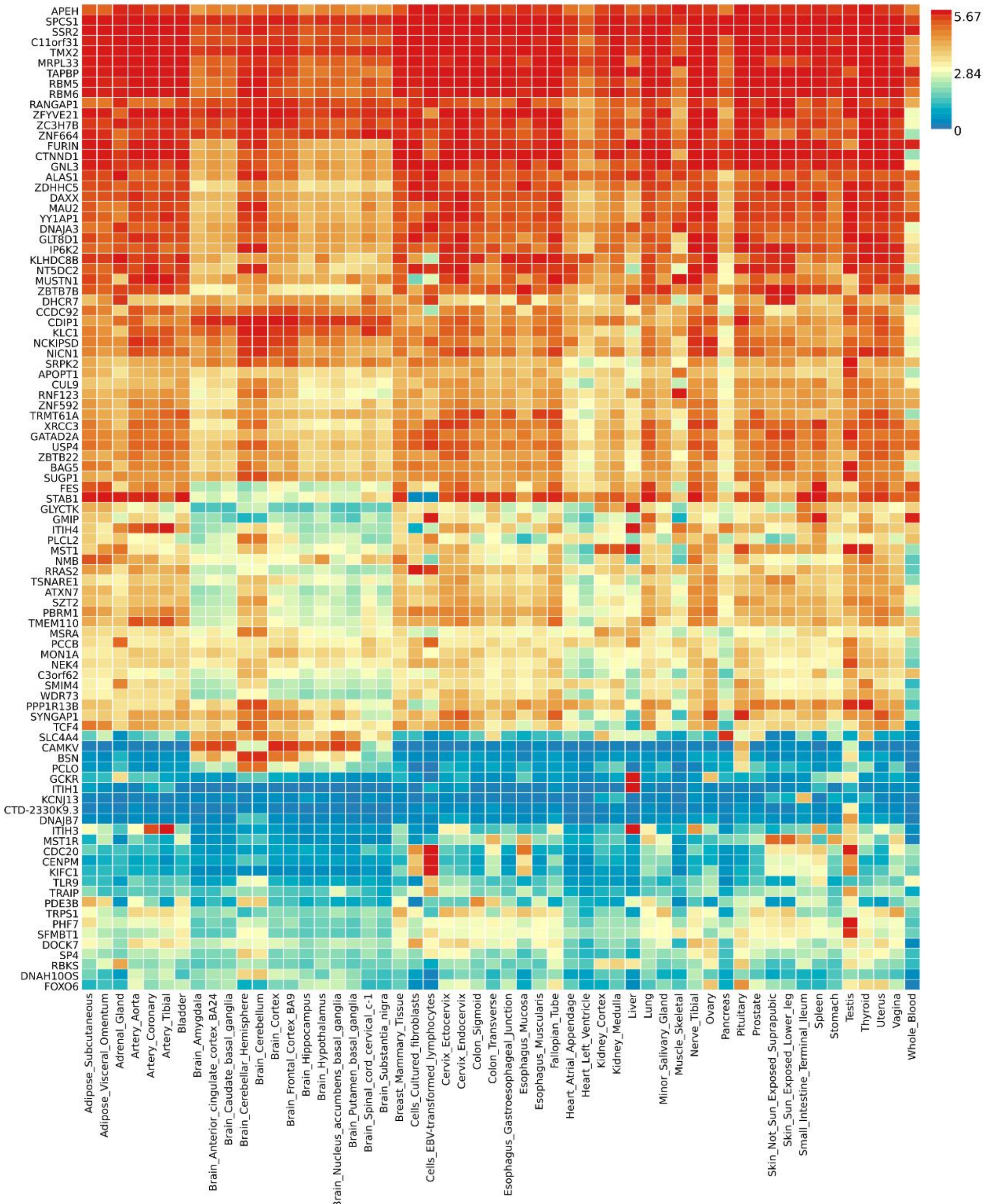

Figure S15. Expression of MAGMA pleiotropic genes in different tissues.

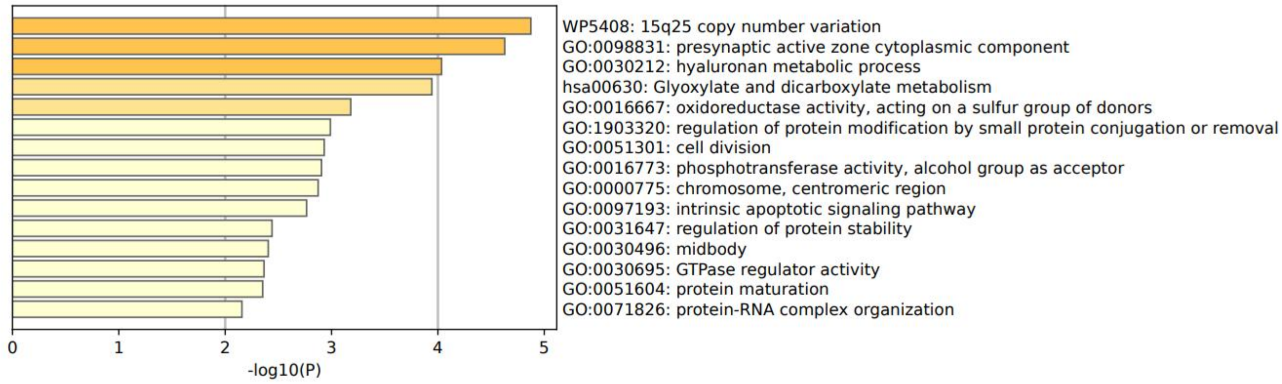

Figure S16. Pathway enrichment of pleiotropic MAGMA genes (KEGG, wiki, GO).

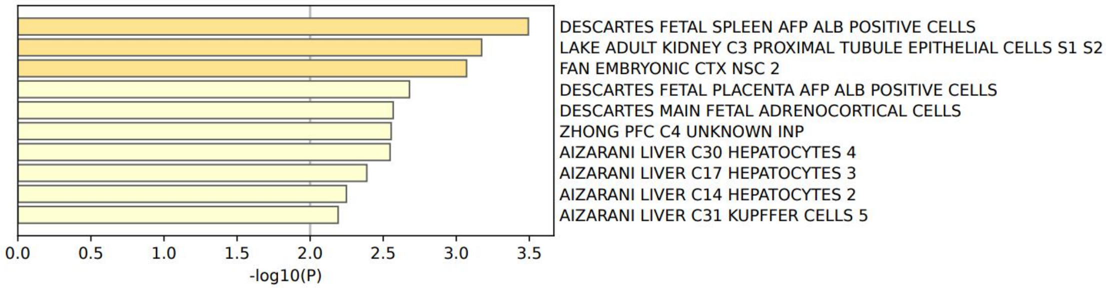

Figure S17. Pathway enrichment of pleiotropic MAGMA genes (cell-type).

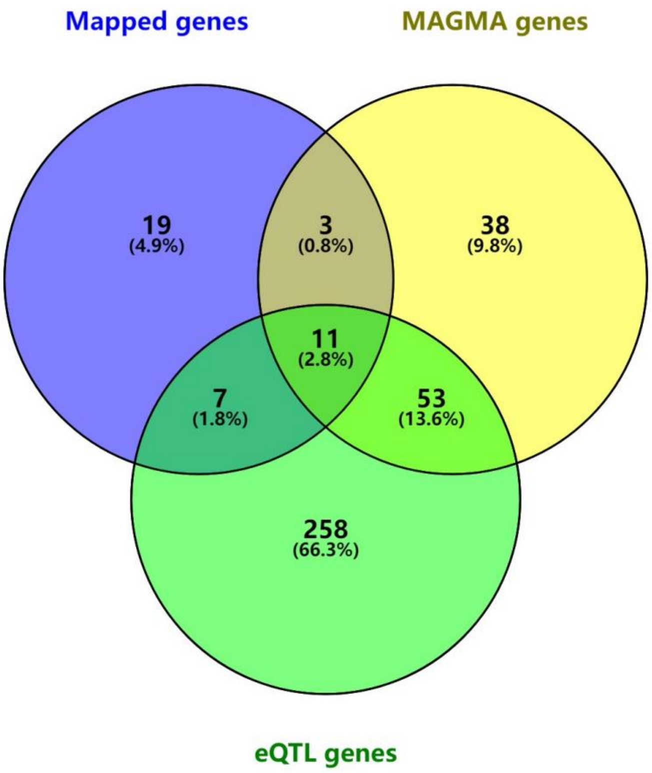

Figure S18. Overlap of pleiotropic genes in different methods.

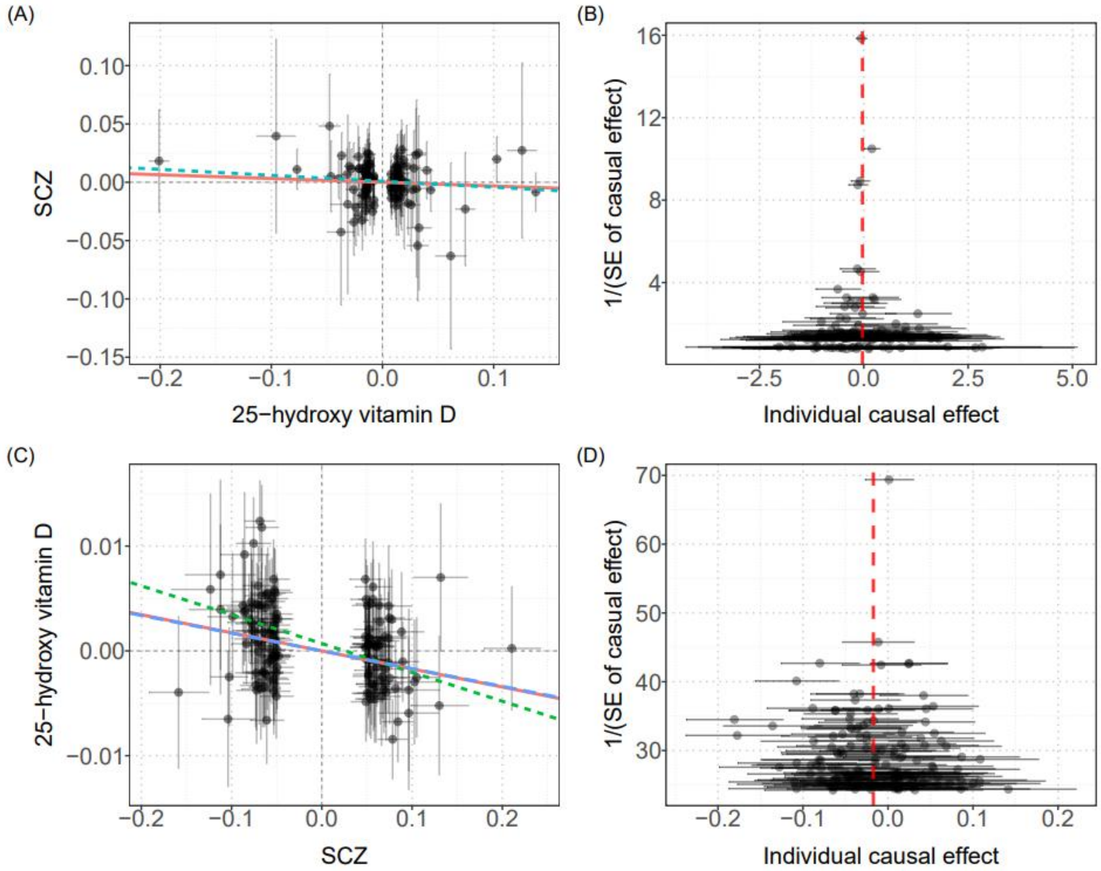

Figure S19. Scatter plot and funnel plot of MR analysis. A is the scatter plot of the causal effect of 25OHD on SCZ; B is the funnel plot of the causal effect of 25OHD on SCZ; C is the scatter plot of the causal effect of SCZ on 25OHD; D is the funnel plot of the causal effect of SCZ on 25OHD.
